# American Medical Association

**Published:** 1873-03

**Authors:** 


### American Medical Association.

The Twenty-fourth Annual Session of the American Medical Association will be held at St. Louis, Mo., May 6th, 1873. The usual committees are expected to report, and many interesting papers will no doubt be presented to the Association. It is to be hoped that none of the disturbing elements of former meetings will be brought up, but that the approaching session will be conducted with that dignity and propriety which should mark all scientific Associations.

The question of a change in the character of the association and in the manner of conducting its meetings has been agitated during the past year, and our brother editors of the *Boston Medical and Surgical Journal*, *The Medical Record*, and *The Chicago Medical Examiner*, have advanced some excellent ideas which if acted on would cause the American Medical Association to be looked upon as a truly scientific body, while in the past some of its meetings have had more of the character of a political caucus.

The following amendments are to be acted upon:

(TO CONSTITUTION·)

*Resolved*, That the United States Marine Hospital Service be placed in the same relative position in the American Medical Association as the Medical Departments of the United States Army and Navy.

And that, in paragraph 2, of the 2d section, after the words "army and navy," the words "and the United States Marine Hospital Service" be inserted.

(TO BY-LAWS.)

SECT. III.—*Standing Committees.*

That, instead of a report on Medical Education, on Medical Literature and

Climatology and Epidemic Diseases, there shall be annually delivered before the Association at its general meetings, an address in Medicine, an address in Surgery, and an address in Midwifery, or the Diseases of Children, the lecturers to be appointed this year by the President; afterwards by the Committee on Nominations.

Also, in section 6, after the words, "the chiefs of the bureaus of the army and navy," be inserted "and the supervising surgeon of the United States Marine Hospital Service."

We shall endeavor to make arrangements whereby we can give our readers in the May Journal a report of the meeting.

Secretaries of all Medical Organization are requested to forward lists of their Delegates, as soon as elected, to the Permanent Secretary, Dr. WM. B. ATKINSON, Philadelphia, Pa.

